# Optical Response Tailoring via Morphosynthesis of Ag@Au Nanoparticles

**DOI:** 10.3390/nano15141125

**Published:** 2025-07-19

**Authors:** David Oswaldo Romero-Quitl, Siva Kumar Krishnan, Martha Alicia Palomino-Ovando, Orlando Hernández-Cristobal, José Concepción Torres-Guzmán, Jesús Eduardo Lugo, Miller Toledo-Solano

**Affiliations:** 1 Facultad de Ciencias Físico-Matemáticas, Benemérita Universidad Autónoma de Puebla, Av. San Claudio y Av. 18 Sur, Col. San Manuel, Ciudad Universitaria, Puebla Pue 72570, Mexico; david.romero@alumno.buap.mx (D.O.R.-Q.); marthap@fcfm.buap.mx (M.A.P.-O.); 2 SECIHTI-Instituto de Física “Ing. Luis Rivera Terrazas”, Benemérita Universidad Autónoma de Puebla, Av. San Claudio y Blvd. 18 Sur, Col. San Manuel, Ciudad Universitaria, Puebla 72570, Mexico; sivakumar@ifuap.buap.mx; 3 Escuela Nacional de Estudios Superiores, Universidad Nacional Autónoma de México, Antigua Carretera a Pátzcuaro 8701, Colonia San José de la Huerta, Morelia 58089, Michoacán, Mexico; ohernandez@enesmorelia.unam.mx; 4 Instituto de Física “Ing. Luis Rivera Terrazas”, Benemérita Universidad Autónoma de Puebla, P.O. Box J-48, Puebla Pue 72570, Mexico; jtorres@ifuap.buap.mx; 5 Faubert Lab, Ecole D’optométrie, Université de Montréal, Montréal, QC H3T 1P1, Canada; 6 Sage-Sentinel Smart Solutions, 1919-1 Tancha, Onna-son Kunigami-gun 904-0495, Okinawa, Japan

**Keywords:** gold@silver nanoparticles, seed-mediated chemical reduction, Maxwell–Garnett theory, multilayer optical model, optical properties

## Abstract

We present a simple method for customizing the optical characteristics of gold-core, silver-shell (Au@Ag) nanoparticles through controlled morphosynthesis via a seed-mediated chemical reduction approach. By systematically adjusting the concentration of cetyltrimethylammonium chloride (CTAC), we obtained precise control over both the thickness of the Ag shell and the particle shape, transitioning from spherical nanoparticles to distinctly defined nanocubes. Bright field and high-angle annular dark-field scanning transmission electron microscopy (BF-STEM and HAADF-STEM), and energy-dispersive X-ray spectroscopy (EDS) were employed to validate the structural and compositional changes. To link morphology with optical behavior, we utilized the Mie and Maxwell–Garnett theoretical models to simulate the dielectric response of the core–shell nanostructures, showing trends that align with experimental UV-visible absorption spectra. This research presents an easy and adjustable method for modifying the plasmonic properties of Ag@Au nanoparticles by varying their shape and shell, offering opportunities for advanced applications in sensing, photonics, and nanophotonics.

## 1. Introduction

Bimetallic NPs comprising a core–shell structure have attracted considerable research interest due to their tunable localized surface plasmon resonance (LSPR) optical properties, which can be varied by modifying not only the size, shape, and shell thickness but also by modulating the composition of both counterparts [1]. These bimetallic core–shell NPs structures hold promise across diverse fields due to the precise control over light manipulation and its interactions with matter. In the field of biomedicine, their tunable optical absorption and scattering render metallic nanoshells applicable in biosensing, imaging, drug delivery, and photothermal therapy [2,3]. In biosensing, LSPR shifts have been exploited to amplify the optical signals of chemical and biological analytes, thereby enhancing detection capabilities [4,5]. Within optics, metallic nanoshells open avenues for phenomena like negative refraction, subwavelength focusing, and cloaking, achieved by manipulating the propagation and scattering of light [6,7]. Effective utilization of these applications demands precise control over metallic nanoshell geometries and accurate theoretical models for their optical absorptions. This is particularly relevant in scenarios such as coupling metallic nanowires to quantum dots, creating plasmonic devices with nanoscale dimensions and minimal losses [8]. Similarly, strategic approaches are employed in optimizing light absorption and photocurrent generation in thin-film solar cells using metallic nanoshells [9]. Moreover, metallic nanoshells find utility in high-density data storage and retrieval by leveraging their multilevel responses to various wavelengths and polarizations of light [10]. Recently, dry-synthesised bi-layer nanoporous metal films have also been reported as versatile plasmonic metamaterials with tunable optical properties [11]. Recent research has also explored the propagation characteristics and bending losses of channel plasmon polaritons (CPPs) in V-shaped grooves with diverse widths and depths using similar plasmonic metamaterial platforms [12]. Additionally, efforts are directed towards demonstrating the fabrication and characterization of channel plasmon polariton-based subwavelength waveguide components operating at telecom wavelengths [13].

Bimetallic NPs with controlled shape and shell thickness are of great importance for plasmonic applications; however, maintaining their size and shape during seed-mediated chemical reduction is a challenging process [14]. Notably, seed-mediated morphosynthesis studies reveal that surfactant- and halide-directed kinetics can steer Au@Ag seeds toward diverse anisotropic architectures, ranging from chemically robust nanodisks to chiral helical nanorods, while core-size engineering modulates catalytic turnover in Au@Ag core–shell systems [15,16]. These insights underscore that fine control over reaction parameters not only tailors LSPR energy and field confinement for advanced plasmonic sensing but also enhances chemical stability, chiroptical activity, and size-dependent catalytic efficiency [16,17,18]. Furthermore, tuning the shapes such as Au@Ag NCs enables tuning of the LSPR properties and their application in surface-enhanced Raman spectroscopy (SERS) due to the presence of sharp corner/edges [19]. Most of the previous studies showed capping agent-free or using polymers [e.g., polyvinylpyrrolidone (PVP)] as the capping agent for controlling the shell thickness and shape of the resultant bimetallic systems [20,21]. However, it is difficult to achieve complete removal of PVP, which has a significant effect on the optical properties and the application of these materials. It was demonstrated that using CTAC as the capping agent enables much greater control over the reduction kinetics to eventually obtain highly uniform core–shell bimetallic NPs with different thickness and shapes [22].

In this work, we demonstrate the controlled synthesis of Au@Ag core–shell nanoparticles by varying the concentration of the surfactant CTAC in the seed-mediated chemical reduction process. In addition, by tailoring the reduction kinetics, such as the Ag-precursor concentration and reduction rate, the Au@Ag NPs can be transformed into Au@Ag core–shell cubes with different thicknesses. The particle size, shape, and core–shell morphology were investigated using various characterization techniques, including scanning transmission electron microscopy in bright field (BF-STEM), high angle annular dark field (HAADF-STEM) modes, and energy-dispersive X-ray spectroscopy (EDS). To achieve the latter, we utilized the Mie theory, which provides insights into the absorption of particles composed of a single material. We extend this theory within the Maxwell–Garnett approximation to enable the determination of the effective dielectric function in cases involving two different materials. This extended framework has found applications in the investigation of NPs characterized by a core that can be either spherical or ellipsoidal, and a shell that may be spherical, ellipsoidal, or cubic in shape. Subsequently, the effective dielectric function derived from this extension is employed to compute the extinction coefficient ($Q_{ext}$), a parameter directly proportional to the UV-Vis absorbance results [23,24].

The analytical model employed in this study, which relies exclusively on the geometrical and optical parameters of metallic nanoshells, provides precise results with significantly reduced computational time compared to conventional numerical methods like finite-difference time-domain (FDTD) or discrete dipole approximation (DDA) simulations [25]. Although these numerical approaches offer accurate solutions, they are computationally demanding and frequently lack straightforward physical interpretation. Our choice of this analytical framework was motivated by the requirement for the efficient prediction of geometric effects (size and shape) on the plasmonic response of synthesized nanoparticles during systematic CTAC concentration optimization. The structure of the paper is as follows: In Section 2, we describe our experimental technique for synthesizing NPs of different thicknesses and shapes. In Section 3, we characterize the morphology, distribution, and size of the obtained samples. In Section 4, we discuss the optical properties of the NPs observed experimentally and compare them with our theoretical model. Finally, in Section 5, we give our conclusions.

## 2. Materials and Methods

### 2.1. Reagents

Gold(III) chloride trihydrate ($HAuCl_{4}$·$3H_{2}O$, ≥99.9%), silver nitrate ($AgNO_{3}$, 99.95%), ascorbic acid (AA, ≥99.0%), sodium borohydride ($NaBH_{4}$, 98%), cetyltrimethylammonium chloride (CTAC, ≥98.0%), and cetyltrimethylammonium bromide (CTAB, ≥98.0%) were purchased from Sigma-Aldrich and were used as received. High-purity deionized water (>18.2 MΩ cm) was collected using a Milipore A10 Mili-Q system and used for all experiments.

### 2.2. Synthesis of Au NPs

The spherical Au NPs with average particle sizes of approximately 7, 20, and 28 nm were prepared following an earlier report [18]. Au seeds of smaller size (2–5 nm) were prepared by mixing 5 mL of 0.1 M CTAB solution, and 50 mM of $HAuCl_{4}$ (100 mM) was added slowly and allowed to be magnetically stirred at room temperature for 5 min to form the Au-CTAB complex. Subsequently, 400 μL of a freshly prepared $NaBH_{4}$ solution was quickly injected under stirring conditions and allowed to react for an additional two minutes to yield the Au seeds. The obtained seeds were stored for further synthesis of Au NPs with a larger particle size.

To prepare ∼7 nm Au NPs, aqueous solutions of CTAC (100 mM, 50 mL), AA (10 mM, 3.25 mL), and the seeds (5 mL) were mixed under magnetic stirring in a 200 mL glass beaker. Then, an aqueous $HAuCl_{4}$ solution (0.5 mM, 20 mL) was added dropwise at a rate of 10 mL/h. The reaction was allowed to continue for 15 min at room temperature after the injection was completed. The final mixture was collected by centrifugation at 15,000 rpm for 30 min and then washed three times with deionized (DI) water to facilitate further growth of the Ag-shell.

To grow ∼20 nm Au NPs directly from the original seeds, aqueous solutions of CTAC (100 mM, 50 mL), AA (10 mM, 3.25 mL), and the seeds (5 mL) were mixed using magnetic stirring in a 200 mL glass beaker. Subsequently, an aqueous solution of $HAuCl_{4}$ (0.75 mM, 20 mL) was added dropwise at a rate of 10 mL/h. After the injection was completed, the reaction was allowed to continue for 15 min at room temperature. The final product was collected by centrifugation at 15,000 rpm for 30 min and washed three times with DI water to yield Au NPs of ∼20 nm.

For the further growth of ∼28 nm Au NPs, the obtained Au NPs (∼10 nm) were dispersed in 10 mL of aqueous solution of CTAC (10 mM). Then, 200 μL aqueous solution of CTAC (60 mM), 250 μL of AA (250 μL) and 10 mL of concentrated pre-synthesized Au NPs (10 nm) solution were mixed and allowed to be magnetically stirred for 15 min. Then, 200 μL of growth $HAuCl_{4}$ (1.0 mM) containing CTAC (100 mM) was slowly injected into the reaction mixture using a syringe pump at an injection rate of 1.0 mL/min. The final products were collected by centrifugation at 15,000 rpm for 30 min and subsequently washed three times to obtain Au NPs of ∼28 nm.

### 2.3. Synthesis of Bimetallic Au@Ag NPs

**Figure 1:**
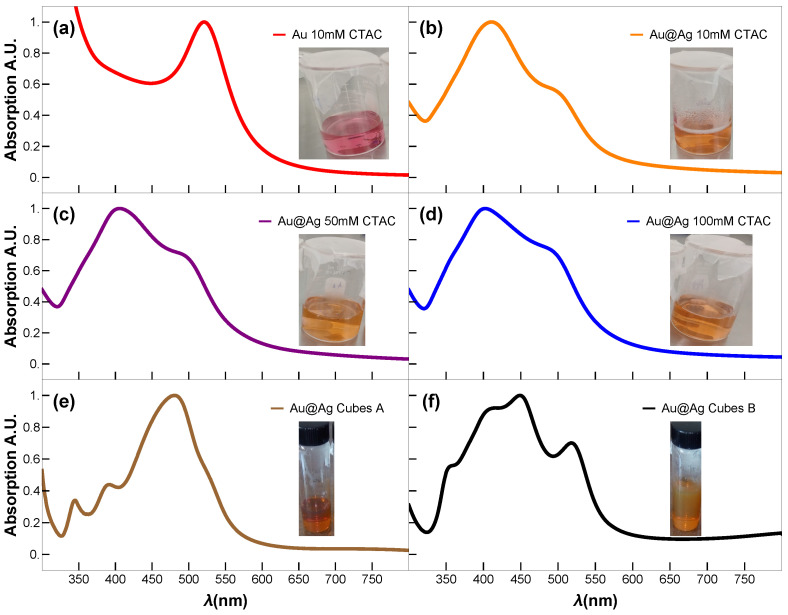
UV-vis absorption spectra of the as-synthesized colloidal Au NP and Au@Ag bimetallic NPs, (**a**) Au seeds of 3 nm diameter, Au@Ag NPs synthesized with (**b**) CTAC 10 mM, (**c**) CTAC 50 mM, (**d**) CTAC 100 mM, Au@Ag NCs (**e**) sample A, and (**f**) sample B, respectively. Insets: show the corresponding photographic images of the colloidal samples.

The synthesis of Au@Ag NPs with varied Ag-shell thickness was prepared by seed-mediated overgrowth of silver shells onto Au NPs cores. In a typical synthesis process, three aqueous solutions of CTAC: 10 mM (50 mL), 50 mM (50 mL), and 100 mM (50 mL), were mixed with the suspension of Au nanospheres (2.5 mL) with a magnetic stirrer at 65 °C in three 50 mL glass beakers, followed by dropwise addition of aqueous $AgNO_{3}$ (25 mM, 200 μL) solution and AA (100 mM, 1 mL) using two micropipettes at an injection rate of 100 μL/h for Au and 1 μL/h for AA, under slow stirring at 65 °C. The reaction was allowed to continue at 65 °C for 15 min after the injection had been finished. Then, the final product was allowed to cool to 27 °C, centrifuged at 14,500 rpm for 45 min, and washed with water. The three colloidal suspensions of Au@Ag nanospheres were dispersed in 10 mL of an aqueous solution of CTAC (10 mM) after washing for further characterization. Figure 1 shows the photographic images of the colloidal Au NPs and Au@Ag core–shell NPs with different shell thicknesses and shapes.

### 2.4. Synthesis of Au@Ag Nanocubes

For preparation of Au@Ag NCs with an edge length ∼40 nm, aqueous solutions of CTAC (100 mM, 10 mL) and the ∼28 nm Au NPs (2.5 mL) were mixed using a magnetic stirrer at 65 °C in a 50 mL glass beaker, followed by dropwise addition of aqueous $AgNO_{3}$ (25 mM, 200 μL) and AA (100 mM, 500 μL) solution using two micropipettes at an injection rate of 100 μL/h for $AgNO_{3}$ and 250 μL/h for AA, under slow stirring at 65 °C. The reaction was allowed to continue at 65 °C for 15 min after the injection had been finished. Then, the final product was allowed to cool to 27 °C, then centrifuged at 15,000 rpm for 30 min and washed with DI water. After that, the final products were dispersed in 10 mL of an aqueous CTAC solution (10 mM) for further characterization after washing. This product is designated as Sample A. The resulting NCs displayed an average edge length of ∼40 nm.

For Sample B, the same procedure was applied using ∼20 nm Au NPs (2.5 mL) in place of the ∼28 nm Au NPs. CTAC (100 mM, 10 mL) and ∼20 nm Au NPs were stirred at 65 °C, then $AgNO_{3}$ (25 mM, 200 μL) was added dropwise at 200 μL/h and AA (100 mM, 500 μL) at 500 μL/h. After 15 min at 65 °C, the mixture was cooled to 27 °C, centrifuged at 18,000 rpm for 30 min, washed with DI water, and then redispersed in 10 mL of 10 mM CTAC.

## 3. Results

STEM images were acquired in a JEM-ARM200F transmission electron microscope operated at an acceleration voltage of 200 kV. Such an analytical microscope is equipped with a spherical aberration corrector, enabling a resolution of 0.82 $\overset{˚}{A}$. For sample preparation, 10 mL of each NP solution was concentrated to 1 mL by centrifugation at 18,000 rpm for 20 min and washed twice to reduce the surfactant concentration below the critical micelle concentration. Subsequently, 10 μL of each solution was drop-casted onto a carbon-coated mesh nickel grid and allowed to dry under ambient conditions. Then, BF and HAADF images were obtained. Furthermore, semi-quantitative measurement of elemental chemistry was carried out with an EDS detector coupled to the microscope. The UV-vis absorption spectrum was acquired using the specimens and the reference (water) through a Cary 5000 spectrophotometer (Varian), which recorded the UV-vis near-infrared (NIR) spectra [26,27,28].

In a typical synthesis process, first Au NPs with an average diameter of 3 nm were prepared in aqueous media by using CTAB as a stabilizing and shape-directing agent and $NaBH_{4}$ as a reducing agent, according to the procedure reported by Haldar [18]. The Au@Ag core–shell nanoparticles with different Ag-shell thicknesses and shapes were achieved through the controlled deposition of Ag atoms over a growth solution containing varied concentrations of CTAC, a stabilizing ligand, and $AgNO_{3}$ as a precursor salt.

**Figure 2:**
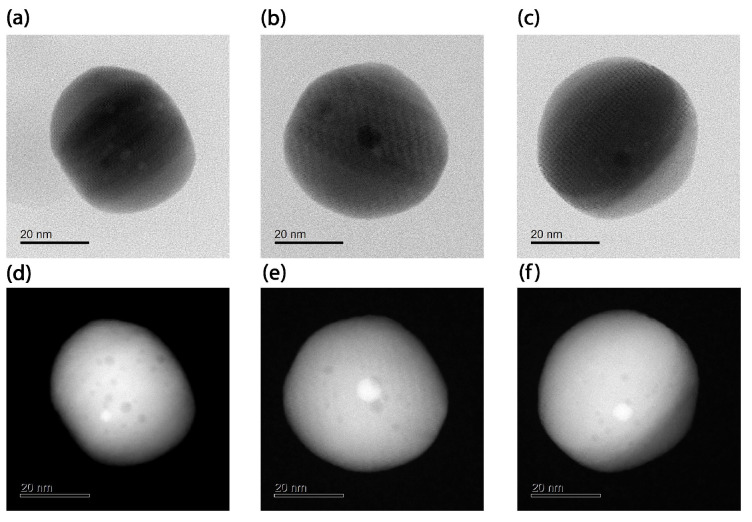
BF-STEM images of Au@Ag NPs, nanospheres synthesized with different CTAC concentrations: (**a**) 10 mM, (**b**) 50 mM, (**c**) 100 mM, and HAADF- STEM of (**d**) 10 mM, (**e**) 50 mM, and (**f**) 100 mM.

**Figure 3:**
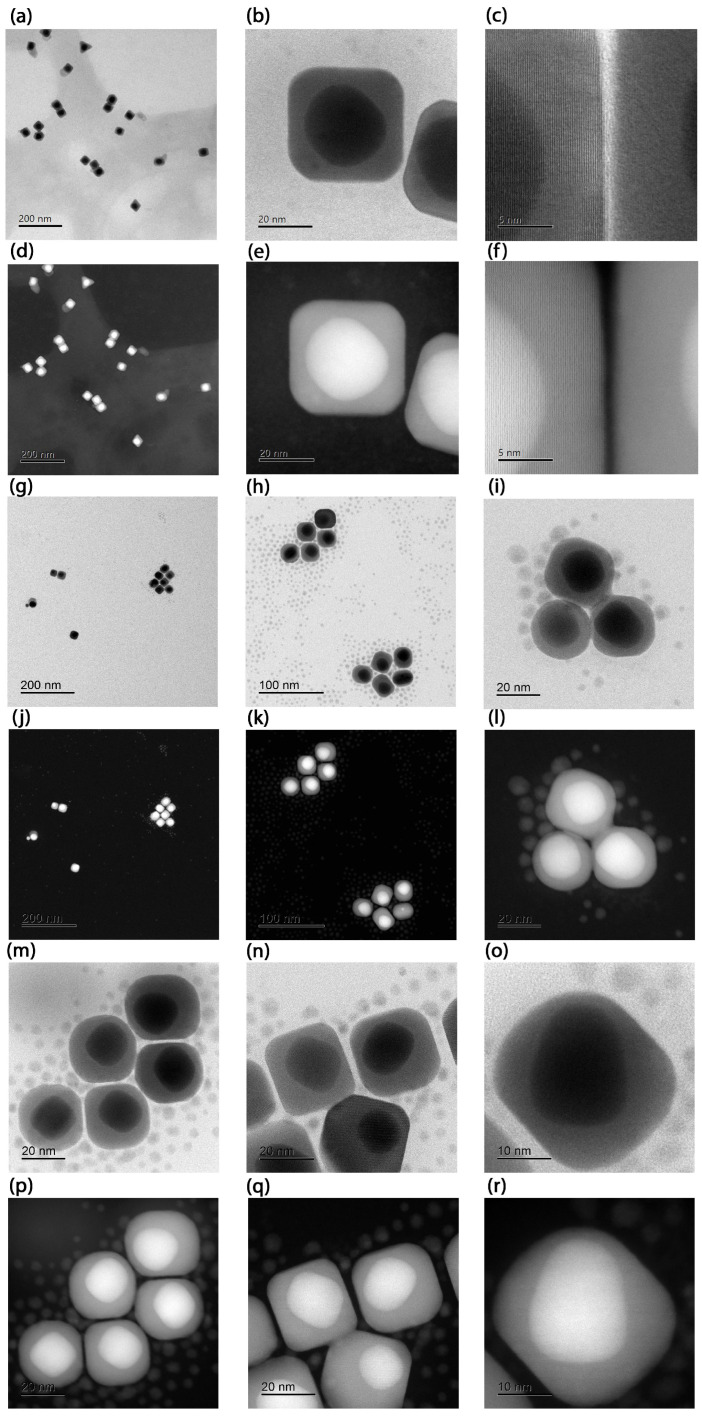
Images of the Sample A with a spherical Au core and a cubic Ag shell: (**a**) BF-STEM image, (**b**) BF-STEM close-up, (**c**) BF-STEM further close-up showing the material transition. In (**d**–**f**) their correspondig images in HAADF-STEM mode. Additional micrographs of Au@Ag NCs from the Sample B: (**g**,**h**) STEM-BF overview and (**i**) further close up. In (**j**–**l**) their correponding images in HAADF-STEM mode. High magnification images in BF-STEM (**m**–**o**) and their corresponding images in HAADF STEM (**p**–**r**) mode.

The BF-STEM and HAADF-STEM images of the synthesized Au@Ag NPs are shown in Figure 2 and Figure 3. As can be seen from Figure 2, the BF-STEM images reveal the formation of a thick Ag-shell onto smaller Au seed nanoparticles. In addition, by observing the HAADF-STEM images in Figure 3, the formation of an Au-core-and-Ag-shell structure was quite evident, as a clear contrast difference was observed between Au and Ag. Furthermore, ImageJ version 1.54f (NIH, USA) software [29] was used to derive the parameters of the Au@Ag nanospheres under the following conditions: (a) using an aqueous solution of 10 mM CTAC, (b) 50 mM CTAC, (c) 100 mM of CTAC and the systems with a spherical Au core and a cubic Ag shell, as shown in Figure 3. The obtained results are summarized in Table 1. The results showed that the Au NPs core had an average diameter of 3 nm, and the Ag shell ranged from 20 to 30 nm. It is important to note that, at a higher CTAC concentration (100 mM), and with increasing Ag precursor salt concentration over the Au NP, the formation of Au@Ag NCs with similar crystallinity to the Au NP core is observed. The high-magnification STEM and HAADF-STEM images in Figure 3 revealed that the formation of highly uniform Au@Ag NCs with uniform shell thickness was achieved. This was confirmed by close observation in high-resolution BF-STEM and HAADF-STEM images (Figure 3) which revealed the Au@Ag core–shell NCs with a clear contrast difference between the spherical Au core–Ag shell. The high-resolution HAADF-STEM images (Figure 3f) confirm the formation of a face-centered cubic (fcc) structure of metallic Ag shell without any lattice mismatch between Au-Ag, which could be due to the almost similar lattice parameters of Au and Ag.

**Figure 4:**
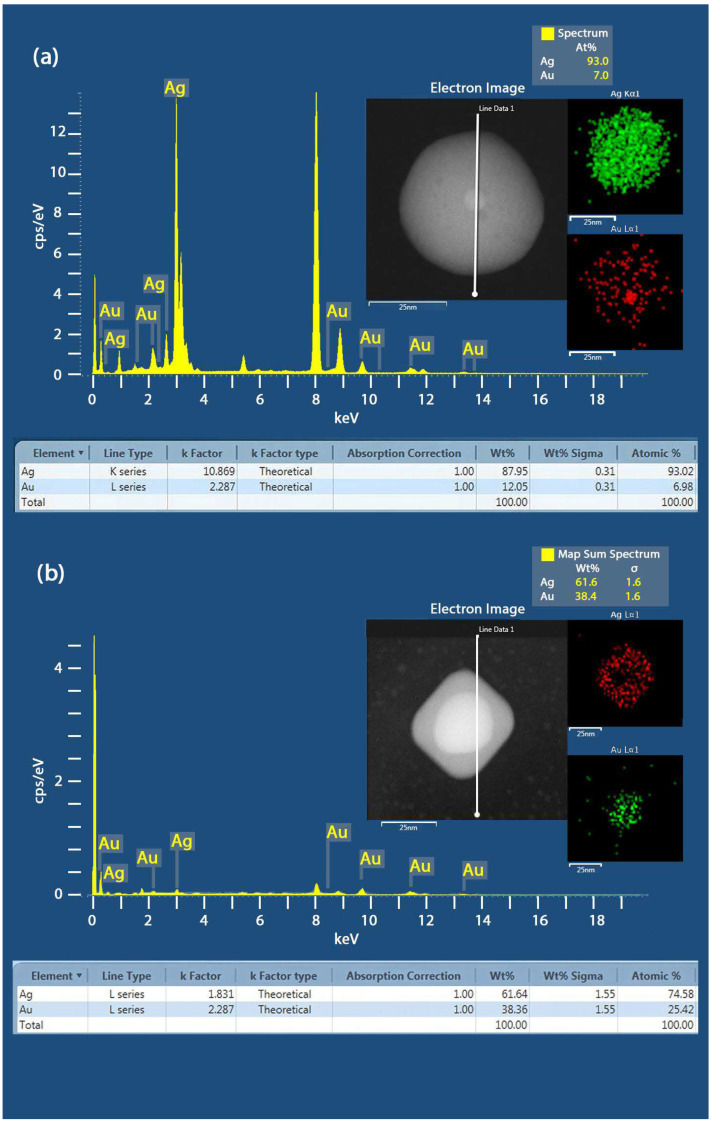
(**a**) EDS spectra of the Au@Ag spherical core–shell NPs samples, showing the element map reconstruction based on the X-ray peaks of Au-Lα and Ag-kα with their eletron image. (**b**) EDS spectra of the Au@Ag NCs Sample A and the element map reconstruction based on the X-ray peaks of Au-Lα and Ag-Lα.

Furthermore, EDS analysis was performed on the spherical Au@Ag samples to determine their elemental composition. As shown in Figure 4a), the EDS spectra of the Au@Ag core–shell NPs revealed the presence of elemental peaks associated with Au and Ag. The composition of Au and Ag was observed to be about 7 and 93 atomic %, suggesting most of the Au core is covered with a thick Ag shell. In the case of Au@Ag NCs, the composition of Au and Ag was about 38.4 and 61.6 atomic %, indicating the formation of a thick Ag shell in the Au@Ag core–shell NCs (see Figure 4).

## 4. Discussion

**Figure 5:**
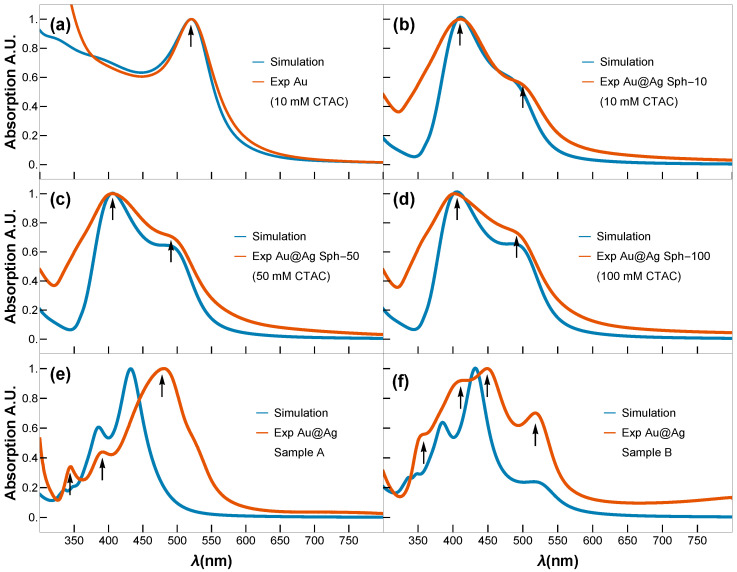
Simulated (blue) and experimental (orange) UV-vis spectra of (**a**) Au seeds (3 nm); (**b**–**d**) Au@Ag core–shell NPs obtained using 10, 50, and 100 mM concentration of CTAC, respectively; (**e**,**f**) Au@Ag NCs Sample A and Sample B. Black arrows indicate LSPR modes.

Figure 5 shows the optical properties of the obtained Au@Ag NPs and Au@Ag NCs determined experimentally by using UV-vis absorption spectroscopy, and the theoretical estimations. Theoretical estimations of the nanostructures optical response were obtained by simulating $Q_{ext}$ (see Appendix A), which is proportional to the UV-vis absorption spectra of the samples, and comparing it with the experimental results (Figure 5). We consider $\hbar \omega_{p}=8.1$ and 6.8 eV for Ag and Au, respectively. The collision time and Fermi velocity were the same for both, namely, $\tau =3.7\times 10^{-14}$ s and $v_{f}=3\times 10^{-14}$ m/s, respectively. The NPs are embedded in water with a refractive index of n=1.33.

Figure 5a displays the expected UV-vis spectrum for small Au NPs, characterized by a single LSPR peak centered around ∼520 nm. In contrast, the UV-vis spectra of the spherical Au@Ag core–shell NPs (Sph-10, Sph-50, Sph-100) display two well-defined LSPR peaks: one at ∼405 nm, attributed to the Ag shell, and a second at ∼490 nm, corresponding to the residual optical contribution of the Au core. Raising the CTAC concentration from 10 to 100 mM produces only a slight increase in the Ag shell thickness ($r_{s}$ grows from ∼20.6 to ∼23.2 nm) while maintaining a highly circular shape (circularity ∼0.9) and essentially constant overall morphology. Consequently, the LSPR band of the Ag shell shows only a minor red shift (Figure 5b–d), despite initial expectations of a more pronounced shift with added Ag content. This limited spectral shift is consistent with classical electrodynamics: a substantially larger red shift typically requires more significant changes in an NP’s effective size or shape, whereas here, the effective geometry remains nearly unchanged. Experimental UV-vis spectra confirm a mild yet systematic red shift of the dipolar plasmon mode with increasing shell thickness, indicating that shell thickening alone (within this narrow ∼3 nm range) imparts only modest tuning of the resonance in a highly symmetric Au@Ag core–shell system. The simulated UV-vis spectra of the plasmonic modes for the Au@Ag core–shell nanospheres closely match the experimentally recorded UV-vis absorption spectra, confirming that the theoretical estimation employed in our study is highly precise.

In contrast, the NCs Samples A and B exhibit distinct plasmonic resonance profiles (Figure 5e,f). While Fuchs’ theoretical model predicts nine LSPRs for perfect NCs [30], only six dominant modes account for >96% of the spectral response. In truncated NCs (generated by corner truncation), LSPRs demonstrate strong morphological dependence [31]. Key trends were observed as blue-shifting of the primary resonance with increasing truncation radius, convergence of shorter-wavelength LSPRs toward the dominant mode (potentially masking them), and broadening of the main LSPR peaks. On the one hand, the cubes of Sample A exhibit minimal truncation; their dipolar and quadrupolar LSPRs are spectrally proximal, merging into a single intense broadband peak centred at ∼480 nm. This peak shows a red shift relative to theoretical predictions, most likely due to experimental conditions involving gold-mediated LSPR coupling—an effect not accounted for in the idealized model. Higher-order multipolar resonances (at shorter wavelengths) show excellent agreement between the Maxwell–Garnett approximation and experimental data. On the other hand, cubes of Sample B display non-uniform gold core distributions (Figure 3m–r), creating thin silver layers that produce a distinct 520 nm peak attributable to gold core excitations. The remaining modes follow the same interpretation as Sample A and match the spectral fingerprints reported for rhombicuboctahedral Ag NCs [30,32], opening avenues similar to those recently reported for nanoporous bi-layer metamaterials [11].

Notably, the results obtained in this study provide insight into the optical behavior of core–shell nanoparticles of varying sizes and shapes, enabling numerous applications in plasmonics. For instance, nanostructures similar to those studied here can be utilized as plasmonic devices for optical fiber communication systems and networks [12,33], as they can enhance the transmission, modulation, and switching of light signals. Tuning the UV–vis absorption into the NIR range enables a broad absorption range in the photocatalysts as well as solar cells. Furthermore, these nanostructures can also function as optical sensors for monitoring the performance and quality of optical networks [34], as they can sense changes in the refractive index, temperature, or strain of the fiber. On the other hand, our model is straightforward and less time-consuming, and it can be easily extended to other plasmonic nanomaterials with different configurations, such as rods, octahedra, or even metamaterials [35].

## 5. Conclusions

In summary, this study demonstrates a tunable approach to tailoring the optical response of Au@Ag core–shell nanoparticles through controlled morphosynthesis. By varying the concentration of CTAC in a seed-mediated chemical reduction process, we achieved precise control over Ag shell thickness and nanoparticle morphology, resulting in the formation of both spherical particles and well-defined nanocubes. High-resolution HAADF-STEM imaging confirmed uniform shell growth and coherent Au–Ag interfaces, free of lattice mismatch. Optical characterization revealed a red shift in the primary Ag LSPR peak (∼405 nm) with increasing shell thickness in nanospheres, while nanocubes exhibited distinct higher-order plasmonic modes. These experimentally observed trends were accurately predicted using the Mie and Maxwell–Garnett models, which provide a computationally efficient framework for understanding and guiding optical tuning better than full-wave approaches such as FDTD and DDA [25]. Overall, our findings highlight a straightforward yet powerful route for engineering the shape-dependent plasmonic properties of AgAu nanoparticles, paving the way for advanced applications in sensing, nanophotonics, and plasmon-enhanced technologies.

## Figures and Tables

**Table 1:** Measured parameters for the Au@Ag NPs. Samples Sph-10, Sph-50, and Sph-100 are Au@Ag nanospheres grown with 10, 50, and 100 mM CTAC, respectively, while Samples A and B are NCs obtained under two distinct growth conditions. Values are expressed as mean ± and standard deviation, calculated from 27 individual particles per sample, measured on three different TEM grids.

Sample	$r_{c}$ (nm)	$r_{s}$ (nm)	Circularity of $r_{c}$ (nm)	Circularity of $r_{s}$
Sph-10	3.10±0.33	20.60±1.73	0.97	0.87
Sph-50	3.60±0.78	23.10±1.02	0.89	0.94
Sph-100	2.75±1.10	23.20±2.50	0.72	0.95
Sample	$r_{c}$ (nm)	Side (nm)	Circularity of $r_{c}$	
Sample A	14.30±0.80	40.40±2.10	0.82	
Sample B	9.10±0.78	31.58±7.60	0.91	

**Figure A1:**
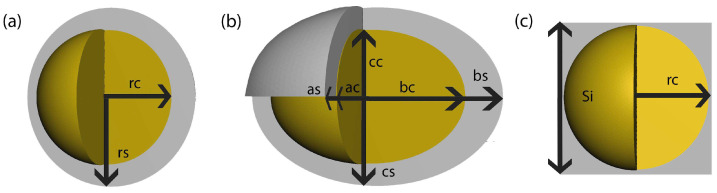
Core–shell systems (**a**) of a core with radius $r_{c}$ and shell with shell radius $r_{s}$, (**b**) of an ellipsoidal core with axes $a_{c}$, $b_{c}$ and $c_{c}$, and ellipsoidal shell with axes $a_{s}$, $b_{s}$ and $c_{s}$ and (**c**) a spherical core with radius $r_{c}$ and cubic shell with side Si.

## Data Availability

The data presented in this study are contained within the article. Additional raw BF-STEM and HAADF-STEM micrographs, together with ImageJ analysis macros, are available from the corresponding author on reasonable request due to their large file size and to avoid redundancy with figures already provided.

## Appendix A. Theoretical Formalism

We investigated the dependence of the complex dielectric function (ϵ) of metallic NPs on the frequency of incident light (ω). Let $\epsilon_{1}$ and $\epsilon_{2}$ be the real and imaginary parts of the dielectric function, respectively. Using the extended Drude model, we considered the reduced mean free path of conduction electrons in NPs smaller than those in the bulk metal. $\epsilon_{1}$ and $\epsilon_{2}$ can be decomposed into two components: one from the bound electrons ($B_{1}$ and $B_{2}$) with discrete energy transitions, and another from the free electrons ($A_{1}\left(\omega \right)$ and $A_{2}\left(\omega \right)$) with a continuous energy distribution. The expressions for these components are provided by

(A1) $$\epsilon \left(\omega \right)=\epsilon_{1}\left(\omega \right)+i\epsilon_{2}\left(\omega \right)=\left[A_{1}\left(\omega \right)+B_{1}\right]+i\left[A_{2}\left(\omega \right)+B_{2}\right],$$

where $A_{1}$ and $A_{2}$ are specified by Equation (A2) and the interband contributions $B_{1}$ and $B_{2}$ are derived from the experimental data of ϵ for the bulk metal by Jhonson and Cristy [36]

(A2) $$A_{1}=1-\frac{\omega_{p}^{2}}{\omega^{2}+\omega_{0}^{2}},\;\;A_{2}=\frac{\omega_{p}^{2}\omega_{0}}{\omega (\omega^{2}+\omega_{0}^{2})}.$$

The plasma frequency $\omega_{p}$ and the collision frequency $\omega_{0}$ are defined as

(A3) $$\omega_{p}=\frac{4\pi N_{e}e^{2}}{m^{*}},\;\;\omega_{0}=\frac{1}{\tau },$$

where τ define the static collision time, $N_{e}$ is the density of the conduction electrons, *e* is the electron charge, and $m^{*}$ is the effective mass of the conduction electron. The product of τ and the Fermi velocity ($v_{f}$) determines the mean free path (*d*) of the conduction electron in the bulk. However, in small particles, this means the free path is shorter due to electron collisions with the particle surface. We account for this effect by adding a term $\tau_{c}$ to the collision time, where $\tau_{c}=d/v_{f}$. This modifies the value of $\omega_{0}$, which affects the dielectric function of the free electrons. So,

(A4) $$\omega_{0}=\frac{1}{\tau }+\frac{v_{f}}{d},$$

using Equation (A1), it is possible to rewrite the dielectric function (ϵ(ω,d)) dependent on the frequency and the mean free path.

The effective dielectric constant of a system composed of NPs embedded in a host medium can be determined using the concept of internal homogenization [37]. This approach enables the derivation of an effective dielectric response, serving as a design tool for tailoring the plasmonic properties of spherical nanoshells. The resulting effective permittivity closely resembles the form given by the Maxwell–Garnett effective medium theory, provided that the core material is treated as an inclusion embedded in a medium of the shell material, where the filling fraction *f* of the core is defined as the core volume divided by the total NP volume—consistent with the core–shell structural configuration. Notably, the Maxwell–Garnett approximation remains valid even for plasmonic mixtures [38], as long as the volume fraction *f* of inclusions remains below 25%, a condition sufficient for accurate permittivity predictions. As evidenced by Table 1, all synthesized NPs in this study exhibit a core filling fraction *f* well below this threshold, confirming the applicability of the Maxwell–Garnett approximation. We obtain the effective dielectric function of the spheres embedded in the host material as follows:

(A5) $$\frac{\epsilon_{ef}-\epsilon_{H}}{\epsilon_{ef}+2\epsilon_{H}}=f\frac{\epsilon_{I}-\epsilon_{H}}{\epsilon_{I}+2\epsilon_{H}},$$

where $\epsilon_{I}$ is the dielectric function of the inclusion embedded in a host of dielectric function $\epsilon_{H}$. Given that for our case, we consider spherical NPs formed by core ($\epsilon_{c}$) as the inclusion and shell ($\epsilon_{s}$) as the host material, solving for the effective dielectric function would result in

(A6) $$\epsilon_{ef}=\epsilon_{s}\frac{\left(\epsilon_{c}+2\epsilon_{s}\right)+2f\left(\left(\epsilon_{c}-\epsilon_{s}\right)\right)}{\left(\epsilon_{c}+2\epsilon_{s}\right)-f\left(\left(\epsilon_{c}-\epsilon_{s}\right)\right)}.$$

Now, we characterize the effective dielectric function of the three core–shell systems: (a) a spherical core and a shell, (b) an ellipsoidal core and shell, and (c) a spherical core and a cubic shell. Figure A1 illustrates a schematic diagram of the proposed system. For the systems (a), (b), (c), the volume fraction *f* depends on the dimensions of the core and the shell; for spheres, it is

(A7) $$f={\left(r_{c}/r_{s}\right)}^{3},$$

where $r_{c}$ is the core radius and $r_{s}$ is the shell radius. For ellipsoids, *f* is given by

(A8) $$f={\left(a_{c}b_{c}c_{c}/a_{s}b_{s},c_{s}\right)}^{3},$$

the corresponding axes of the core are $a_{c}$, $b_{c}$, and $c_{c}$, and of the shell are $a_{s}$, $b_{s}$, and $c_{s}$, and for the spherical core with cubic shell, *f* is given by

(A9) $$f=\frac{4\pi r_{c}^{3}/3}{S_{i}^{3}},$$

with a core radius $r_{c}$ and a cubic shell side $S_{i}$.

We can utilize the effective dielectric function in the Mie theory, along with the reflection coefficients for Au and Ag, which depend on the particle size (radius). For the core–shell case, we also consider the value *d* [36,39,40], which is the characteristic distance of the system given by d=4V/S, where *V* is the volume of the particle and *S* is the surface between the core ($s_{c}$) and the shell ($s_{s}$). For the system (a), consisting of spheres, this is given by

(A10) $$d=\frac{4(r_{s}^{3}-r_{c}^{3})}{3(r_{s}^{2}+r_{c}^{2})},$$

for system (b), ellipsoids, this is given by

(A11) $$d=\frac{16\pi (a_{c}b_{c}c_{c}-a_{s}b_{s}c_{s})/3}{s_{c}+s_{s}},$$

where $s_{c}$ and $s_{s}$ denote the values of the function *s* for the core and the shell, respectively. The function *s* depends on three parameters *a*, *b*, and *c*, which vary for the core and the shell. Thus, we write $s_{c}=s\left(a_{c},b_{c},c_{c}\right)$ and $s_{s}=s\left(a_{s},b_{s},c_{s}\right)$. The expression of the function s is given by

(A12) $$s=2\pi c^{2}+\frac{2\pi ab}{\sin(\phi )}(E\left(\phi ,k\right)\sin^{2}\left(\phi \right)+F\left(\phi ,k\right)\cos^{2}\left(\phi )\right),$$

where

(A13) $$\begin{array}{cc} & \cos\left(\phi \right)=\frac{c}{a},\;k^{2}=\frac{a^{2}\left(b^{2}-c^{2}\right)}{b^{2}\left(a^{2}-c^{2}\right)},\;a\geq b\geq c,\;0\leq \phi \leq \frac{\pi }{2}, \end{array}$$

F(ϕ,k) and E(ϕ,k) being the first and second order elliptic integrals [41].

Finally, for the system (c), we have to obtain the dielectric function that separates the geometric properties from the dielectric properties [39]. Therefore, the dielectric function will depend on weighted factors $C_{n}$ that satisfy $\Sigma_{n}C_{n}=1$, the depolarization factor of a nanosphere $L_{i}=1/3$, and $L_{n}$ a real number in the interval [0,1]. Each $L_{n}$ is associated with the n-th depolarization factor of an NC, and $C_{n}$ is its weight factor; these data are taken from Table A1 [39]. Hence, the dielectric function is composed of the weighted average of all the modes, each mode with its weight factor $C_{n}$,

(A14) $$\begin{array}{ccc} & \epsilon_{ef}=\sum_{n}C_{n}\epsilon_{ef}^{\left(n\right)}, & \\ & \epsilon_{ef}^{\left(n\right)}=\epsilon_{c}\left(1+\frac{f(\epsilon_{n}-\epsilon_{c})}{\epsilon_{c}+\left(\epsilon_{n}-\epsilon_{c}\right)\left(L_{i}-fL_{n}\right)}\right). \end{array}$$

We have obtained the effective dielectric function of the two-material NPs, taking into account their interband, intraband, size, and volume fraction contributions. Now, we use the scattering efficiencies ($Q_{ext}$) [23,42] that include the quadrupole plasmon resonances in terms of the effective dielectric function of the NPs and the medium [43].

**Table A1:** Table of the six main modes of the cube *n*, weighted factors $C_{n}$ and their depolarization factors $L_{n}$.

*n*	$C_{n}$	$L_{n}$
1	0.44	0.214
2	0.24	0.297
3	0.04	0.345
4	0.05	0.440
5	0.10	0.563
6	0.09	0.706

The electric field of light causes the conduction electrons in a metallic particle to oscillate in unison. This is the dipole plasmon resonance of the particle. There are also higher modes of plasmon excitation, like quadrupolar modes. We use the quasistatic approximation to assume that the electric field is constant over time. We also need the effective dielectric function of the particle (ϵ) and the medium ($\epsilon_{0}$). The incident electromagnetic wave has an electric field $E_{0}$. We take this vector in the x-direction, so $E_{0}=E_{0}\hat{x}$, where $\hat{x}$ is a unit vector. To find the electromagnetic field near the particle, we solve the Laplace equation, $\nabla^{2}\varphi =0$, where ϕ is the electric potential and E=−∇φ. We use two boundary conditions: (1) φ is continuous at the particle surface, and (2) the normal component of the electric displacement D=ϵE is also continuous.

The Laplace equation has spherical harmonics as angular solutions. The radial solutions are of the form $r^{l}$ and $r^{-(l+1)}$, where *l* is the angular momentum label (l=0,1,2,…) of the atomic orbitals. For the l=2 solution, and $E_{0}$ in the x-direction, the potential inside the sphere (r<d) is φ=Arsinθcosϕ and outside the sphere (r>d) is $\varphi =(-E_{0}r+B/r^{2})\sin\theta \cos\phi$, where A and B are constants. Using the boundary conditions and φ, we obtain the field outside the sphere $E_{out}$ as

(A15) $$\begin{array}{cc} E_{out}= & E_{0}\hat{x}-\alpha E_{0}\left[\frac{\hat{x}}{r^{3}}-\frac{3x}{r^{5}}\left(x\hat{x}+y\hat{y}+z\hat{z}\right)\right]\\ \; & +i\frac{2\pi }{\lambda }E_{0}\left(x\hat{x}+z\hat{z}\right)-\beta E_{0}\left[\frac{x\hat{x}+z\hat{z}}{r^{5}}-\frac{5z}{r^{7}}\left(x^{2}\hat{x}+y^{2}\hat{y}+xz\hat{z}\right)\right], \end{array}$$

where the first term is the applied field, the second is the dipole field from the electron polarization, and the last is the quadrupole term. $\hat{x}$, $\hat{y}$, $\hat{z}$ are unit vectors. α is the sphere polarizability, and β is the quadrople polarizability, respectively, given by

(A16) $$\begin{array}{cc} \alpha = & g_{d}d^{3},\\ \beta = & g_{q}d^{5}, \end{array}$$

where

(A17) $$\begin{array}{cc} g_{d}= & \frac{\epsilon -\epsilon_{0}}{\epsilon +2\epsilon_{0}},\\ g_{q}= & \frac{\epsilon -\epsilon_{0}}{\epsilon +3/2\epsilon_{0}}. \end{array}$$

Finally, using the Mie theory [42], we can derive the quasi-static (dipole + quadrupole) equation of the extinction efficiency as

(A18) Qext=4xdIm[gd+xd212gq+xd230(ϵ−1)],xd=2πdϵ01/2/λ.
